# Supplementary material for: Bacterial Metabolite Indole Modulates Incretin Secretion from Intestinal Enteroendocrine L Cells
Source: Cell Rep. 2014 Nov 13;9(4):1202–8. doi: 10.1016/j.celrep.2014.10.032 (PMC4308618; doi:10.1016/j.celrep.2014.10.032)
Supplement: Document S1. Figure S1 [file mmc1.pdf]

Cell Reports, Volume *9*

Supplemental Information

Bacterial Metabolite Indole Modulates Incretin Secretion from Intestinal Enteroendocrine L Cells

Catalin Chimerel, Edward Emery, David K. Summers, Ulrich Keyser, Fiona M. Gribble, and Frank Reimann

Supplementary Information

Bacterial metabolite indole modulates incretin secretion from intestinal enteroendocrine L-cells.

Catalin Chimerel, Edward Emery, David K. Summers, Ulrich Keyser, Fiona M. Gribble and Frank Reimann

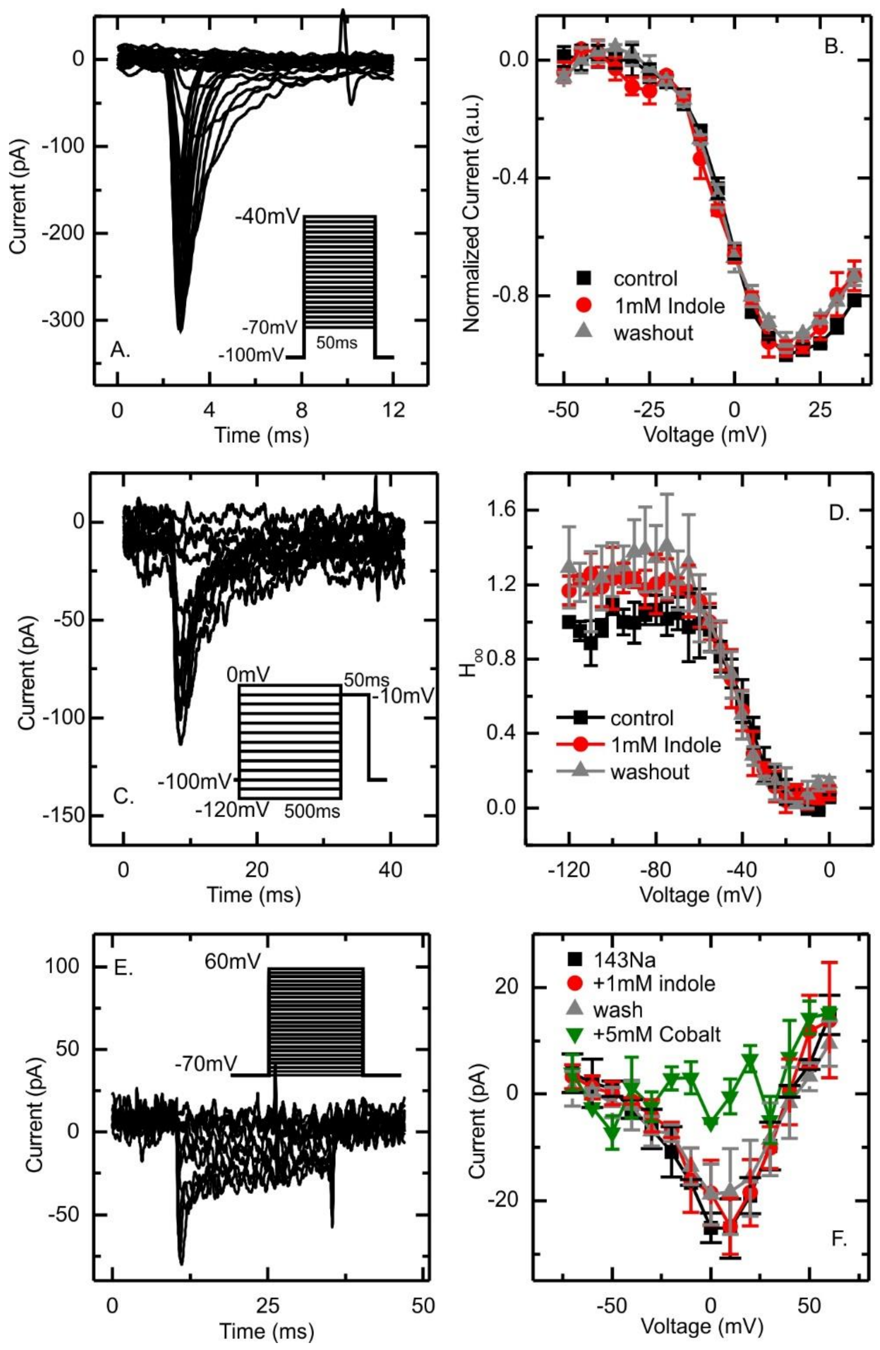

Supplementary Figure 1. **Effect of indole on sodium and calcium currents in GLUTag cells, related to Figure 2. A.** Current responses to 50 ms voltage steps of 5 mV increments were

applied from a holding potential of -100 mV (shown in the inset) applied in a whole cell voltage clamp recording in saline buffer containing inhibitors of voltage gated K^+ channels (see material and methods). Recordings were done in a whole cell voltage clamp recording where 50 ms voltage steps of 10 mV increments were applied from a holding potential of -100 mV. The raw data shown in panel A were down-sampled from 10 kHz to 1 kHz with a digital low pass filter (Clampfit 10.3.1.4). **B.** Current voltage relationship of the peak currents obtained when activating voltage gated Na^+ channels as in A. Each individual experiment was normalized to the maximum peak current at + 15 mV control. Data show are mean ± SEM of n=3 cells. **C.** Steady-state inactivation of Na^+ currents measured by holding the membrane at -100 mV for 500 ms at conditioning voltages between -120 and 0 mV (at 5 mV increments) before stepping to -10 mV for 50 ms and then to -100 mV. The inset shows the voltage pulse protocol. Only the current reponse to the test pulse to -10 mV is shown. The raw data shown in panel A were down-sampled from 10 kHz to 1 kHz with a digital low pass filter (Clampfit 10.3.1.4). An electrical interference Filter (Clampfit 10.3.1.4) was applied to remove the power line interference at 50 Hz. **D.** Mean peak currents from cells recorded as in C, normalized to the maximum peak current at -120 mV control for each individual experiment (H_∞), and plotted against the holding potential applied during the conditioning pules. Data show are mean ± SEM of N=3 cells. **E.** Current responses to 25 ms voltage steps of 5 mV increments, applied from a holding potential of -70 mV (shown in the inset), in a whole cell voltage clamp recording. Voltage gated Na^+ and K^+ current were blocked with specific inhibitors (see material and methods). The raw data shown in panel A were down-sampled from 10 kHz to 1 kHz with a digital low pass filter (Clampfit 10.3.1.4) . An electrical interference Filter (Clampfit 10.3.1.4) was applied to remove the power line interference at 50 Hz. **F.** Current voltage relationship of the peak currents obtained from cells recorded as in A (N=3).
