# Supplementary material for: Bacterial Metabolite Indole Modulates Incretin Secretion from Intestinal Enteroendocrine L Cells
Source: Cell Rep. 2014 Nov 13;9(4):1202–8. doi: 10.1016/j.celrep.2014.10.032 (PMC4308618; doi:10.1016/j.celrep.2014.10.032)
Supplement: Document S2. Article plus Supplemental Information [file mmc2.pdf]

Cell Reports

Bacterial Metabolite Indole Modulates Incretin Secretion from Intestinal Enteroendocrine L Cells

Graphical Abstract

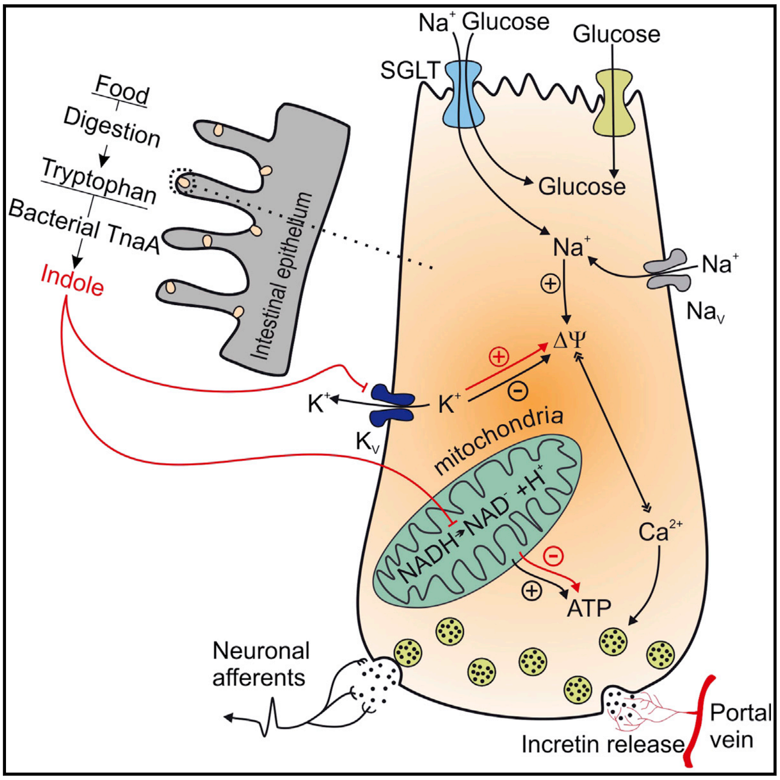

Authors

Catalin Chimerel, Edward Emery, ..., Fiona M. Gribble, Frank Reimann

Correspondence

cc539@cam.ac.uk (C.C.),
fmg23@cam.ac.uk (F.M.G.),
fr222@cam.ac.uk (F.R.)

In Brief

Indole is the main metabolite produced by gut bacteria from tryptophan. Chimerel et al. demonstrate that indole modulates the hormone secretion of enteroendocrine L cells and reveal the molecular mechanism behind this modulation. These findings suggest that the production of indole by bacteria could have a major impact on host metabolism.

Highlights

Bacterial metabolite indole modulates secretion of incretin peptide GLP-1

Indole widens the width of action potentials fired by L cells and elevates GLP-1

Prolonged exposure to indole inhibits ATP production and thus GLP-1 secretion

Chimerel et al., 2014, Cell Reports 9, 1202–1208
November 20, 2014 ©2014 The Authors
http://dx.doi.org/10.1016/j.celrep.2014.10.032

OPEN ACCESS
CellPress

Cell Reports
Report

Bacterial Metabolite Indole Modulates Incretin Secretion from Intestinal Enteroendocrine L Cells

Catalin Chimerel,[1,2,4,*] Edward Emery,[1,4] David K. Summers,[3] Ulrich Keyser,[2] Fiona M. Gribble,[1,*] and Frank Reimann[1,*]
[1]Institute of Metabolic Science and MRC Metabolic Diseases Unit, University of Cambridge, Addenbrooke's Hospital, Hills Road, Cambridge CB2 0QQ, UK
[2]Cavendish Laboratory, Department of Physics, University of Cambridge, JJ Thomson Avenue, Cambridge CB3 0HE, UK
[3]Department of Genetics, University of Cambridge, Downing Street, Cambridge CB2 3EH, UK
[4]Co-first author
*Correspondence: cc539@cam.ac.uk (C.C.), fmg23@cam.ac.uk (F.M.G.), fr222@cam.ac.uk (F.R.)
http://dx.doi.org/10.1016/j.celrep.2014.10.032
This is an open access article under the CC BY license (http://creativecommons.org/licenses/by/3.0/).

SUMMARY

It has long been speculated that metabolites, produced by gut microbiota, influence host metabolism in health and diseases. Here, we reveal that indole, a metabolite produced from the dissimilation of tryptophan, is able to modulate the secretion of glucagon-like peptide-1 (GLP-1) from immortalized and primary mouse colonic L cells. Indole increased GLP-1 release during short exposures, but it reduced secretion over longer periods. These effects were attributed to the ability of indole to affect two key molecular mechanisms in L cells. On the one hand, indole inhibited voltage-gated K^+ channels, increased the temporal width of action potentials fired by L cells, and led to enhanced Ca^{2+} entry, thereby acutely stimulating GLP-1 secretion. On the other hand, indole slowed ATP production by blocking NADH dehydrogenase, thus leading to a prolonged reduction of GLP-1 secretion. Our results identify indole as a signaling molecule by which gut microbiota communicate with L cells and influence host metabolism.

INTRODUCTION

Obesity is one of the biggest health and socioeconomic issues of the 21st century, with the number of obese individuals worldwide almost doubling within the last 30 years. People suffering from obesity are at high risk of developing other metabolic diseases such as type 2 diabetes mellitus (T2DM), cardiovascular disease, and nonalcoholic fatty liver disease (Nicholson et al., 2012). Increasing physical activity and reducing food intake are recommended when treating obesity and associated metabolic diseases, but outcomes are only moderately successful as such lifestyle changes are not sustained long-term by most patients. Alternative strategies therefore need to be developed in order to control body weight more effectively. Anorectic gut hormones such as glucagon-like peptide 1 (GLP-1) and peptide YY are secreted into the circulatory system by enteroendocrine L cells in response to changes in the content of the gut lumen and are at the frontline of the search for new therapies. Gut hormones play essential roles in a wide range of metabolic functions such as the regulation of food absorption, appetite, and glucose homeostasis. As a consequence, drugs that enhance GLP-1 action are now widely used in the treatment of T2DM (Garber et al., 2009; Nauck et al., 2009; Zinman et al., 2009) and are under investigation for the treatment of obesity (Marre et al., 2009). Attention is also turning toward the enteroendocrine L cells themselves and whether they could be targeted for the treatment of obesity and diabetes.

Enteroendocrine L cells are distributed along the length of the intestinal epithelium and thus make direct contact with the gut microbiota. The colon not only harbors the highest density of enteroendocrine L cells within the intestine, but it is also host to the largest number of bacteria. Although it is widely believed that gut microbiota can modulate the function of colonic L cells, our understanding of the molecular mechanisms underlying this potential crosstalk is limited (Cani et al., 2013; Devaraj et al., 2013). Among the metabolites produced by bacteria in the gut, recent attention has focused on short-chain fatty acids, produced by the fermentation of unabsorbed starch and nonstarch polysaccharides. Short chain fatty acids activate G protein coupled receptors (GPCRs) expressed on the plasma membrane of L cells, enhancing L cell number and secretion (Cani et al., 2013; Petersen et al., 2014; Plaisancié et al., 1995; Psichas et al., 2014; Tarini and Wolever, 2010; Tolhurst et al., 2012). Many other bacterial metabolites are also abundantly present within the luminal contents of the colon (Nyangale et al., 2012). Here we focused on a metabolite of tryptophan, indole, as it has recently been shown that plasma tryptophan concentrations are decreased in mice following Roux-en-Y gastric bypass surgery (Mutch et al., 2009) and elevated in people with a high risk of developing T2DM (Wang et al., 2011). Moreover, indole production has been shown previously to vary with the dietary tryptophan content in human subjects (Bryan, 1966).

Indole, the most prevalent metabolite of tryptophan, is produced by a wide range of bacterial species, including those belonging to the genera Escherichia, Bacteroides, and Clostridium (DeMoss and Moser, 1969; Lee and Lee, 2010; Smith and Macfarlane, 1996). These bacterial species use tryptophanase to degrade tryptophan into indole, pyruvate, and ammonia,

CrossMark

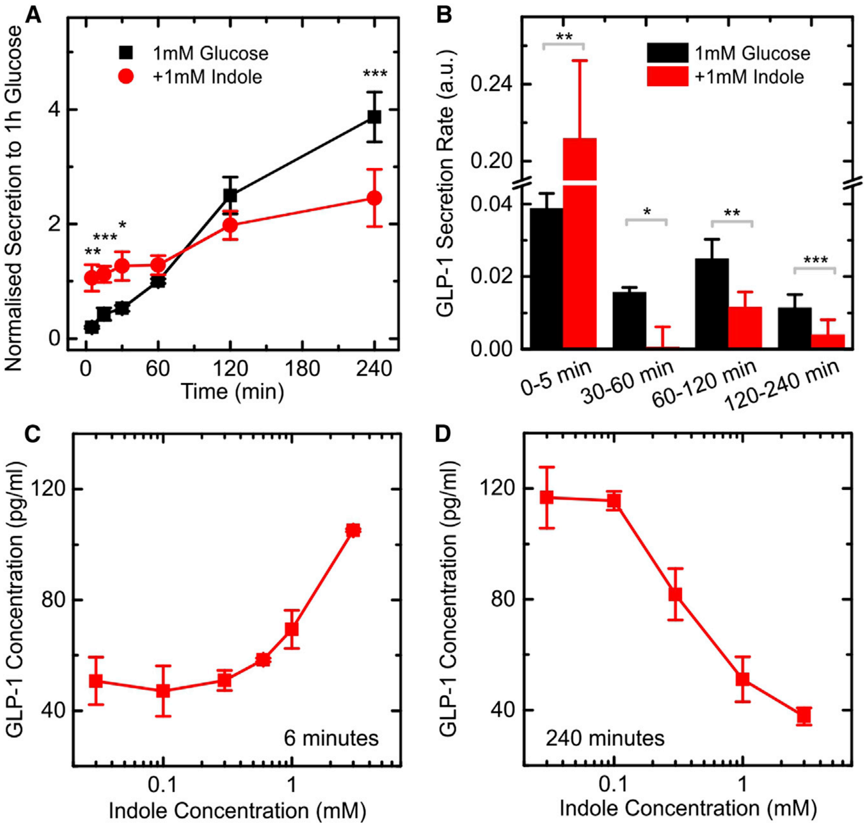

Figure 1. Modulation of GLP-1 Secretion in GLUTag Cells by Indole

(A) Cumulative GLP-1 secretion from GLUTag cells stimulated with 1 mM glucose, measured at different time points. Cells were incubated with and without 1 mM indole, and secretion was normalized to the 60 min, 1 mM glucose, time point (average value of 15 pg/ml) from the same experiment. Each data point was calculated by averaging over eight or more independent measurements. Significance was calculated at specific time points comparing the secretion at 1 mM glucose and the secretion at 1 mM glucose + 1 mM indole.

(B) Calculated rates of GLP-1 secretion over the time periods indicated, calculated from the data in (A). a.u., arbitrary units.

(C) GLP-1 secretion measured in response to different indole concentrations for an incubation time of 6 min in the presence of 1 mM glucose (measured from three independent measurements). To gain resolution the data were measured on a plate with high cell density (45 pg/ml GLP-1 secreted after 6 min in 1 mM glucose).

(D) GLP-1 secretion measured in response to different indole concentration for an incubation time of 240 min on a plate with high cell density (measured from three independent measurements). Data are presented as mean ± SEM. $^*p < 0.05$, $^{**}p < 0.01$, $^{***}p < 0.001$ by paired Student's t test. The lines between points are drawn to guide the eye.

which leads to an average indole concentration ranging from 0.25 to 1.2 mM in human faeces (Karlin et al., 1985; Zuccato et al., 1993). Indole has been previously identified as a beneficial signaling molecule involved in crosstalk between the microbiota and gut wall by increasing epithelial cell tight junctions and attenuating indicators of inflammation (Bansal et al., 2010). Here we show that the bacterial signal indole modulates GLP-1 secretion from enteroendocrine cells, and we identify the molecular mechanisms underlying this interaction.

RESULTS

Indole Modulates GLP-1 Secretion from GLUTag Cells

Cumulative GLP-1 secretion from GLUTag cells was measured in the presence of saline buffer containing 1 mM glucose and 0.1% (wt/vol) BSA over 240 min (Figure 1A). The GLP-1 concentration in the medium increased approximately linearly with time under these conditions, reflecting a relatively constant GLP-1 secretory rate (Figure 1B). In the additional presence of 1 mM indole, GLP-1 secretion was modified over both short and long time periods. Very high GLP-1 concentrations were measured in the medium of indole-treated cells after only 5 min, indicating a rapid initial stimulation of secretion (Figures 1A and 1B). Thereafter, GLP-1 concentrations continued to rise, but the rate of release was slower than that observed in the absence of indole (Figure 1B). At the end of the 240 min incubation period, cumulative released GLP-1 was ~35% less in the presence of 1 mM indole than in its absence. These data indicate that although indole acutely stimulates GLP-1 secretion, it exerts a suppressive effect on the rate of hormone release over prolonged time periods. We examined the dose dependence of indole on both the short- and long-term actions by measuring cumulative GLP-1 release after 6 and 240 min incubations (Figures 1C and 1D). After 6 min, the acute stimulatory effect of indole was evident at concentrations above 1 mM. After 240 min, by contrast, the suppressive effect was evident at indole concentrations as low as 0.3 mM. To clarify the interaction between indole and GLUTag cells, we investigated the molecular mechanisms that determine the modulation of GLP-1 secretion by indole.

Indole Modifies the Action Potential Waveform

GLUTag cells fire action potentials, which are coupled to influx of extracellular calcium and the exocytosis of GLP-1 loaded vesicles (Gribble et al., 2003). We investigated the effect of indole on action potential firing using whole-cell current clamp recordings of GLUTag cells stimulated by injection of a series of 20 ms current pulses of increasing amplitude (Inset of Figure 2A). In the presence of 1 mM indole, we observed that the temporal width of the action potential was increased almost 2-fold (Figures 2A and 2B). This was associated with a slower rate of membrane repolarization (Figure 2A), but no apparent change in the rate of membrane depolarization during the action potential upstroke (Figure 2A).

OPEN
ACCESS
CellPress

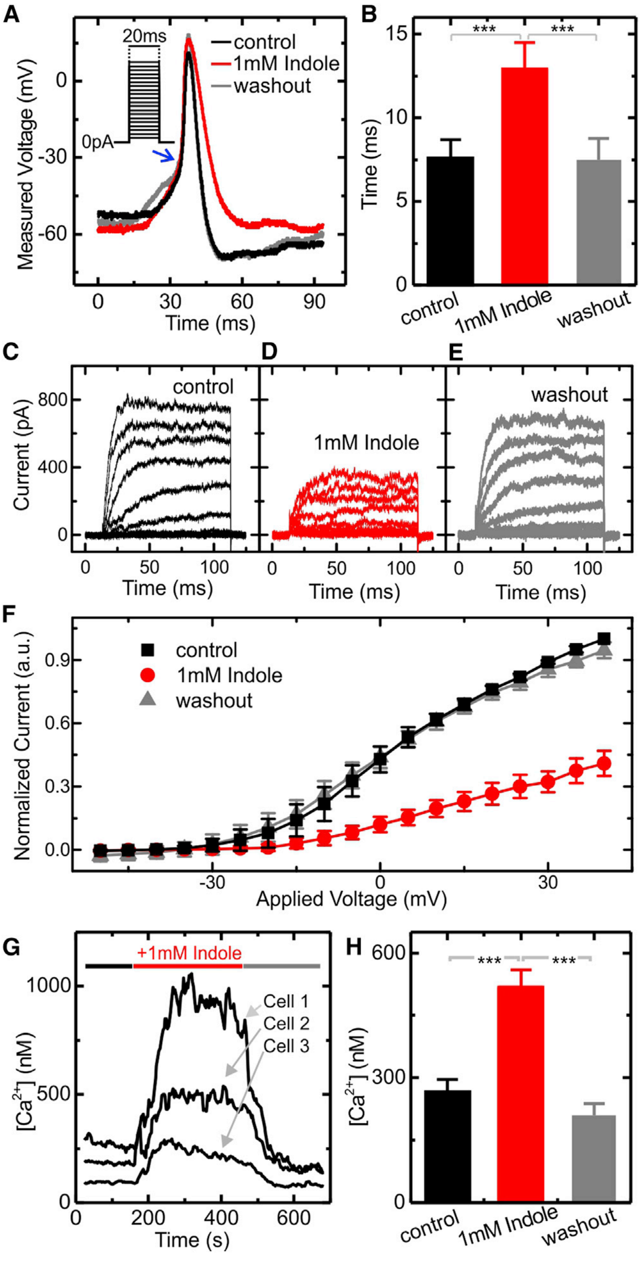

Figure 2. Reshaping of the Action Potential by Indole

(A) Representative traces of action potentials fired by GLUTag cells measured in whole-cell patch current clamp recordings. Current was injected to maintain the cell at ~−55 mV, and the action potentials were stimulated by injection of 2 ms depolarizing currents of increasing magnitude (in steps of 2 pA) as shown in the inset. Recordings were made in standard bath solution, before and during perfusion with 1 mM indole, and after indole washout.

(B) The width of the action potential measured at the threshold of the steep action potential upstroke (as indicated by the blue arrow in (A). Values are means of 5 or more independent measurements. Inhibition of K^+ currents in GLUTag cells by indole.

(C–E) Representative voltage clamp traces from GLUTag cells recorded using the whole-cell patch clamp configuration in saline buffer containing 0.3 μM TTX before (C), during (D), and after (E) the addition of 1 mM indole.

(F) Steady-state current voltage relationships averaged over five different experiments. Cells were maintained at a holding potential of −70 mV, and a series of square wave voltage pulses at 5 mV increments (between −50 and +40 mV) was applied at 0.2 s intervals. Currents were normalized to the control measured at +40 mV. Increased intracellular Ca^{2+} concentration in the presence of indole.

(G) Calcium concentrations measured in single GLUTag cells, recorded using fura-2AM. The 1 mM indole was perfused as indicated by the horizontal bar. Three representative traces are shown.

(H) Mean data from 77 cells recorded as in (G), before and during 1 mM indole perfusion, and after indole washout.

All data are presented as mean ± SEM. *p < 0.05, **p < 0.01, ***p < 0.001 by Student's t test.

Indole Inhibits Voltage-Gated K^+ Channels

To analyze which ionic currents underlie the slower rate of membrane repolarization, we isolated and measured the voltage-gated K^+ and Na^+ currents, which are the key components involved in action potential firing. Ionic currents through voltage-gated K^+ channels were measured using whole-cell patch clamp electrophysiology, in the presence of the voltage-gated Na^+ channel inhibitor, tetrodotoxin (TTX; 0.3 μM) (Figures 2C–2F). We observed that the K^+ currents were inhibited more than 50% by 1 mM indole (Figure 2F). Voltage-gated Na^+ and Ca^{2+} currents, by contrast, were not affected by the presence of indole (Figure S1 available online). These data strongly suggest that indole widens the action potentials by reducing the amplitude of the K^+ current, thus slowing membrane repolarization.

Indole Increases the Intracellular Concentration of Ca^{2+} Ions in GLUTag Cells

We next investigated whether the altered action potential waveform results in changes in the concentration of intracellular Ca^{2+} ions. Ca^{2+} was measured upon exposure of GLUTag cells to 1 mM indole, using the ratiometric dye Fura-2 AM. Indole was found to result in a rapid and reversible elevation of intracellular Ca^{2+} (Figures 2G and 2H). As the properties of voltage-gated Ca^{2+} currents were not themselves influenced by indole, the elevation of intracellular Ca^{2+} is likely to arise from the widened action potentials, resulting in the cell spending more time in a depolarized state.

Indole Reduces ATP Production

As indole was found to reduce the rate of GLP-1 secretion during long-term exposures (30–240 min; Figure 1B), we investigated its effects on cellular metabolism. Indole is known to affect mitochondrial ATP production by blocking NADH dehydrogenase and by facilitating proton permeation ("uncoupling") through the mitochondrial membrane (Chimerel et al., 2013). To monitor NAD(P)H levels in GLUTag cells, we excited the intrinsic fluorescence of NAD(P)H molecules at 360 nm (±15 nm). In the presence of indole, NAD(P)H autofluorescence was observed to increase (Figure 3A), as would be expected when the oxidation of NADH to NAD^+ is blocked. As a positive control, we used 1 μM rotenone, which is an established blocker of NADH dehydrogenase. Rotenone, like indole, resulted in an increase in NAD(P)H autofluorescence. We also assessed the ability of indole to block oxidative phosphorylation, by monitoring the intracellular ATP/ADP ratio using the genetically encoded sensor, Perceval, which was transiently transfected into GLUTag cells (Berg et al., 2009; Tarasov et al., 2012). Addition of indole to the extracellular medium

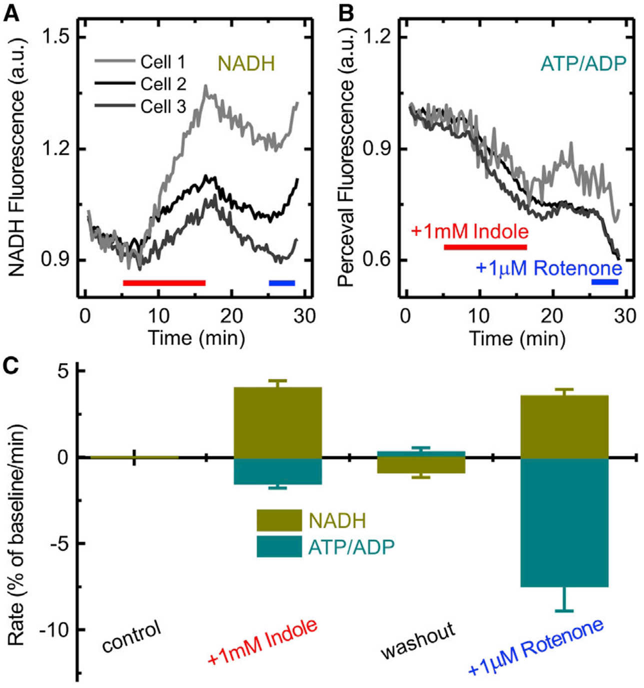

Figure 3. Effect of Indole on NAD(P)H and ATP/ADP Ratio

(A and B) Representative traces of NAD(P)H autofluorescence (A) and the ATP/ADP ratio monitored by Perceval fluorescence (B), in three individual GLUTag cells. The 1 mM indole was added to the perfusion solution, as indicated by the red bars, and 1 μM rotenone was perfused as indicated by the blue bars.

(C) Mean rates of change in the signals for NAD(P)H and ATP/ADP ratio calculated during addition of either 1 mM indole or 1 μM rotenone. The rate measured during the control (in the presence of saline plus 1 mM glucose) is set to zero by subtracting it from the rates measured at 1 mM indole, washout and 1 μM rotenone for each individual cell. In the graph the rates are the means ± SEM for 27 cells.

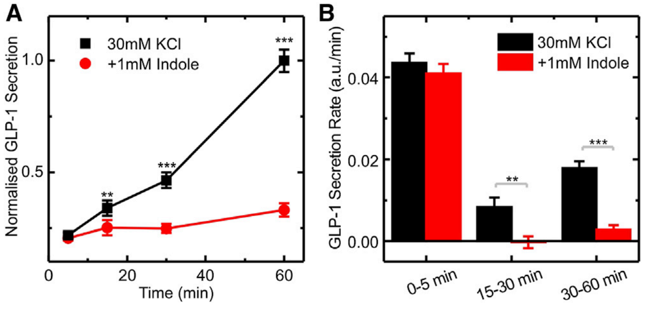

Figure 4. Effect of Indole on KCl Stimulated GLP-1 Secretion

(A) Cumulative GLP-1 secretion from GLUTag cells stimulated with 30 mM KCl, measured at different time points. Cells were incubated with and without 1 mM indole, and secretion was normalized to the 60 min, 30 mM KCl time point (average value 232 pg/ml) from the same experiment. Each data point was calculated by averaging over six independent measurements.

(B) Calculated rates of GLP-1 secretion over the time periods indicated, calculated from the measurements obtained in (A).

Data represent the mean ± SEM. **p < 0.01, ***p < 0.001 by Student's t test.

resulted in a decrease in the Perceval fluorescence intensity, indicative of a fall in the intracellular ATP/ADP ratio (Figure 3B). Perceval fluorescence decreased with a rate of 1.5% ± 0.3%/min in the presence of 1 mM indole and with a rate of 7.5 ± 1.5%/min in 1 μM rotenone. These data suggest that indole affects intracellular ATP generation. In light of this result, we hypothesize that the observed inhibitory effect of indole on GLP-1 secretion over longer time periods is a consequence of the lowered intracellular ATP concentration in GLUTag cells.

Influence of Indole on KCl-Triggered GLP-1 Secretion from GLUTag Cells

We showed previously that GLP-1 secretion by GLUTag cells is strongly stimulated by increasing the extracellular K^+ concentration, which results in membrane depolarization and voltage-gated Ca^{2+} entry (Friedlander et al., 2011; Reimann et al., 2005). At the same time this is an ATP-demanding process, as energy is required to sustain the high rate of GLP-1 release. When GLUTag cells were incubated in the presence of 30 mM KCl, GLP-1 secretion increased 13-fold compared with that measured from cells in 4.5 mM KCl, as measured by cumulative secretion over 60 min. As evident from Figure 4, 1 mM indole did not affect cumulative GLP-1 secretion within the first 5 min of exposure to KCl, but reduced cumulative release after 15 min by 25% and after 60 min by ~70%. When these data were represented as the rates of secretion during different time intervals, it was evident that indole did not affect the GLP-1 secretory rate during the first 5 min of exposure to 30 mM KCl, but drastically reduced secretion in the 5–15 min interval and beyond.

Effects of Indole on Primary L Cells

To investigate whether the effects of indole on GLUTag cells are relevant to native colonic L cells, we repeated some of the key experiments in primary murine colonic cultures. The effect of indole over short time periods was tested by analyzing its effect on intracellular Ca^{2+} levels in L cells, measured using a genetically encoded calcium sensor GCaMP3 specifically targeted to the L cell population in a transgenic mouse model. Intracellular Ca^{2+} levels in primary L cells increased upon exposure to 1 mM indole (Figures 5A and 5B), confirming that the electrophysiological effects of indole recorded in GLUTag cells are likely also to be relevant in the native L cell population. To test for a longer-term suppressive effect of indole on cellular metabolism, we measured GLP-1 release from primary colonic cultures after a 2 hr incubation period. This was reduced by ~50% in the presence of 1 mM indole (Figure 5C), consistent with the similar effects observed in GLUTag cells.

DISCUSSION

Our observations demonstrate that GLP-1 secretion from enteroendocrine cells is modified by exposure to indole at concentrations similar to those found in the human large intestine. Interestingly, indole enhanced the rate of GLP-1 release over short exposure times but slowed secretion over longer time periods. Our data suggest that the production of indole by the gut microbiota could have a major impact on host metabolism, presenting indole as a bacteria-derived signaling molecule capable of modulating enteroendocrine cell function.

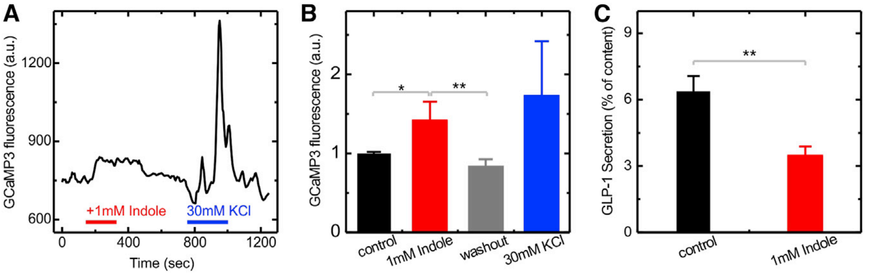

Figure 5. Effects of Indole on L Cells in Primary Murine Colonic Cultures

(A) Representative trace showing the GCaMP3 fluorescence, reporting the intracellular Ca^{2+} level, in a primary colonic L cell perfused with 1 mM indole and 30 mM KCl, as indicated by the horizontal bars.

(B) Mean GCaMP3 fluorescence from L cells, recorded as in (A), before and during indole perfusion, after indole washout, and during perfusion with 30 mM KCl. Values are means ± SEM for nine cells.

(C) GLP-1 release measured from primary colonic cultures incubated for 2 hr in the presence of 10 mM glucose, with and without 1 mM indole, as indicated. Each data point is calculated by averaging over six independent measurements.

$*p < 0.05$, $**p < 0.01$ by Student's t test.

We identified distinct molecular mechanisms underlying the stimulatory and inhibitory effects of indole on GLP-1 secretion. Over short time periods, indole widened action potentials by reducing the current passing through voltage-gated potassium channels. This resulted in prolonged activation of voltage-gated Ca^{2+} channels and translated into an enhanced rate of GLP-1 release. Consistent with the widened action potentials, intracellular Ca^{2+} levels were approximately doubled in the presence of 1 mM indole. It is interesting to note that the effects of indole on intracellular Ca^{2+} and action potential waveform were fully reversed following indole washout. This is consistent with previous findings that indole diffuses freely through lipid membranes (Piñero-Fernandez et al., 2011) and shows that indole does not bind irreversibly to the cell membrane or ion channels. Supporting our observations, inhibition of voltage-gated potassium channels by similar concentrations of indole has been reported in other biological models (Avdonin and Hoshi, 2001), suggesting that indole could affect a range of different excitable cell types even if their complement of K^+ channels is not identical. It is therefore not surprising that the stimulatory effect of indole on Ca^{2+} concentrations in GLUTag cells was translatable to L cells in primary colonic cultures. Although GLP-1 secretion from primary colonic L cells over less than 30 min is difficult to measure by immunoassay because GLP-1 concentrations are at the lower end of the assay range, increases in intracellular Ca^{2+} are an established marker of L cell activity (Tolhurst et al., 2011) and are likely to translate to an enhancement of GLP-1 release over short time periods. When GLUTag cells were activated with 30 mM KCl, GLP-1 secretion increased ~15-fold compared with saline buffer containing only 4.5 mM KCl (42 pg/ml versus 2.5 pg/ml after 5 min), but was not further enhanced by indole. This was not surprising, as 30 mM KCl depolarizes the cells considerably beyond the activation threshold of voltage-gated Ca^{2+} channels (~ -40 mV), and any widening of action potentials would become irrelevant.

Over longer time periods, indole acted as an inhibitor of NADH dehydrogenase and an uncoupler of mitochondrial oxidative phosphorylation, resulting in a lowered intracellular ATP concentration in GLUTag cells. One action of reduced ATP levels would be to open ATP-sensitive K^+ (K_{ATP}) channels in GLUTag-cells, thereby hyperpolarizing the plasma membrane and slowing GLP-1 release (Reimann and Gribble, 2002). While this might contribute to the inhibitory effect of indole on cells treated with 1 mM glucose, it cannot account for the suppression by indole of GLP-1 secretion at high extracellular K^+ concentrations, because under these conditions a larger K^+ current would rather tend to promote K^+-dependent membrane depolarization and calcium entry. An alternative potential explanation is that when mitochondrial ATP generation is suppressed by indole, there is insufficient energy to support vesicle exocytosis and replenishment, particularly when high secretory rates need to be sustained over long periods.

Interestingly, indole exerted an inhibitory effect on GLP-1 release at slightly lower concentrations (0.3 mM) than those found to significantly enhance secretion (1 mM). Which of these actions of indole dominates in vivo is unclear, but it is possible that low indole concentrations cause a tonic suppression of secretion from colonic L cells, with the balance changing in favor of GLP-1 stimulation after a high protein meal. An acute stimulatory effect on GLP-1 secretion could contribute to the observed inhibition of appetite when rats were fed on a high protein diet as compared with carbohydrate and fat (Aziz and Anderson, 2003; Peters et al., 2001). Expression of the tryptophanase enzyme in most bacteria is induced in the presence of tryptophan and repressed by glucose (Gong et al., 2001). High protein diets would therefore tend to promote, and high sugar diets suppress, indole production. Moreover, it has been shown that protein diets with high tryptophan content are better at suppressing appetite, and it was speculated that this effect was due to the conversion of tryptophan to serotonin, a neurotransmitter involved in the suppression of appetite (Aziz and Anderson, 2003). Here we have identified another possible mechanism for this inhibition, involving the production of indole, which stimulates GLP-1 secretion over short time periods. On the other hand, our measurements suggest that long-term feeding on a moderate to high protein diet could have an inhibitory effect on GLP-1 production as ATP levels in L cells could be affected. In the future the amplitudes, time fluctuations, and spatial variation of indole production should be investigated in the luminal content of the gut to better understand the crosstalk between the microbiota and the host.

EXPERIMENTAL PROCEDURES

GLP-1 Secretion

All animal procedures were approved by the local ethics review committees and conformed to UK Home Office regulations. GLP-1 secretion was assessed by immunoassay from both GLUTag (Drucker et al., 1994) and mouse colonic L

cells in mixed intestinal epithelial cultures plated as previously described (Reimann and Gribble, 2002). In brief, cells were plated in 24-well plates and cultured overnight. On the day of the experiment, cells were washed twice with 400 μl of standard saline buffer (4.5 mM KCl, 143.5 mM NaCl, 2.6 mM $CaCl_2$, 1.2 mM $MgCl_2$, and 10 mM HEPES [pH 7.4 with NaOH]) supplemented with 1 mM glucose and 0.1% (wt/vol) BSA. Experiments were performed by incubating the cells with the saline buffer complemented with the corresponding indole (analytical standard grade, Sigma Aldrich) amount diluted from a 500 mM stock in ethanol. For GLUTag cell secretion measurements, the GLP-1 secreted into the supernatant was quantified. For primary cells, secretion was expressed as a fraction of the total hormone content (secreted + extracted) measured from a well (Reimann et al., 2008). GLP-1 concentrations were measured using a total GLP-1 assay (Mesoscale Discovery). For the maximal stimulation of GLP-1 secretion from GLUTag cells, the KCl concentration in the secretion media was raised to 30 mM while reducing the NaCl to keep constant osmotic concentrations.

Electrophysiology

GLUTag cells were patch clamped and monitored as previously described (Reimann et al., 2005). Voltage-gated K^+, Na^+, Ca^{2+} currents, and the firing of action potentials were assessed. In brief, GLUTag cells were plated on 35 mm dishes 1–2 days prior to patching. Microelectrodes were pulled from filamented borosilicate glass capillaries (GC150TF-15, Harvard Apparatus), and the tips were coated with yellow beeswax melted at 90°C. The tip of the microelectrode was fire polished using a microforge (MF-830, Narishige) and had a resistance of 2–4 MΩ when filled with patch solution. Membrane potentials and currents were measured with an Axopatch 200B amplifier (Molecular Devices) through a Digidata 1440A digitiser (Molecular Devices) and processed with pCLAMP 10.3 software (Molecular Devices). The acquisition frequency was set to 10 kHz (low-pass Bessel filter), while the sampling frequency was 25 kHz or higher. For Na^+ and Ca^{2+} currents the standard whole-cell currents were leak subtracted using pCLAMP 10.3 software protocol P/4.

The standard whole-cell patch pipette solution contained 107 mM KCl, 1 mM $CaCl_2$, 7 mM $MgCl_2$, 11 mM EGTA, 10 mM HEPES, and 5 mM Na_2ATP (pH 7.2 with KOH). The standard bath solution was the same as above. For measurement of K^+ currents, bath solution contained saline buffer + 0.3 μM TTX and the pipette solution contained 107 mM KCl, 1 mM $CaCl_2$, 7 mM $MgCl_2$, 11 mM EGTA, 10 mM HEPES, and 5 mM K_2ATP (KOH to pH 7.2). For measurement of Na^+ currents, the bath solution contained 115 mM NaCl, 2.6 mM $CaCl_2$, 5 mM $CoCl_2$, 1.2 mM $MgCl_2$, 5 mM CsCl, 20 mM TEA-Cl_2, and 10 mM HEPES (NaOH to pH 7.4) and the pipette solution contained 107 mM CsCl, 5 mM $MgCl_2$, 11 mM EGTA, 10 mM HEPES, and 5 mM NaATP (CsOH to pH 7.2). Similar solutions were used to measure Ca^{2+} currents, with the exception that $CoCl_2$ was omitted from, and 0.3 μM TTX was added to, the bath solution.

Ca^{2+} Imaging

Imaging of Ca^{2+} levels in response to indole was performed in both GLUTag and mouse colonic L cells. GLUTag cells were plated on matrigel-coated 35 mm glass bottom dishes, 1–2 days prior to use and loaded with fura-2-acetoxymethyl ester (fura-2-AM; Invitrogen). A standard saline buffer with 1 mM glucose added was used as a control measurement. The dish was mounted in a perfusion chamber on an Olympus IX71 microscope with ×40 oil-immersion objective and imaged using an Orca-ER CCD camera. A 75W Xenon arc lamp and a monochromator (Cairn Research) controlled by MetaFluor software (Universal Imaging) were used to alternately excite the dye at 340(10) and 380(4) nm. The Fura-2-AM fluorescence was measured at >510 nm. Free cytoplasmic Ca^{2+} concentrations were estimated for individual cells from the ratio of 340/380 nm fluorescence intensities assuming a K_D of 224 μM for fura-2-AM (Grynkiewicz et al., 1985). At the end of the experiment 5 μM ionomycin in 5 mM EGTA was used to calibrate for 0 mM Ca^{2+} and 5 μM ionomycin in 5 mM Ca^{2+} used to calibrate for the maximal response to Ca^{2+}.

For mouse colonic L cells, the calcium levels were measured using a mouse line obtained by crossing GLU-Cre mice (Parker et al., 2012) with commercially available Rosa26-GCamp3 reporter mice (Jax strain 014538) (Zariwala et al., 2012), resulting in expression of the genetically encoded Ca^{2+}-sensor in L cells. The fluorescence of the Ca^{2+}-sensor was excited with 488(10) nm and the emission was measured at 535(35) nm. A total of 36 colonic L cells were imaged, of which 9 responded to indole and 13 to KCl. From the nine cells responding to indole, five did respond to KCl.

NADH and Perceval Imaging

Imaging of NAD(P)H levels in GLUTag cells was performed by monitoring the intrinsic fluorescence emission of the cell at >510 nm when excited at 360(15) nm. For determining the cytoplasmic ATP/ADP ratio Perceval plasmid (Berg et al., 2009; Tarasov et al., 2012) was transfected into GLUTag cells. Its fluorescence was excited with 490(2) nm and measured at >510 nm. The NAD(P)H and Perceval were monitored in parallel by alternatively switching between the excitation wavelengths. The same optical setup and experimental settings as for Ca^{2+} imaging was used. A standard saline buffer with 1 mM glucose added was used as a control measurement.

Data Analysis

The average values are calculated as means ± SEM. The paired Student's t test was used in Figure 1 (only) because of a large variance in the basal GLP-1 secretion in the absence of indole as a consequence of different plating densities used when plating GLUtag cells on the secretion plate. Otherwise, significance was tested by two-sample equal variance Student's t test, and the significance intervals were considered as follows: *0.05 > p > 0.01, **0.01 > p > 0.001, ***0.001 > p.

SUPPLEMENTAL INFORMATION

Supplemental Information includes one figure and can be found with this article online at http://dx.doi.org/10.1016/j.celrep.2014.10.032.

AUTHOR CONTRIBUTIONS

C.C., U.F.K., F.M.G., and F.R. initiated the research project. C.C. and E.E. performed the experiments; all authors analyzed and discussed the data. C.C., F.M.G., and F.R. designed the experiments and wrote the manuscript.

ACKNOWLEDGMENTS

C.C. and U.F.K. acknowledge the European Research Council (ERC) grant (261101 PassMembrane) for funding. F.M.G., F.R., and E.E. were funded by the Wellcome Trust (WT088357/Z/09/Z and WT084210/Z/07/Z) and the MRC Metabolic Diseases Unit (MDU), Cambridge. GLP-1 immuno-assays were performed by Keith Burling at the MRC-MDU. GLUTag cells were kindly provided by Dr. D. Drucker (Toronto, Canada).

Received: July 29, 2014
Revised: September 22, 2014
Accepted: October 10, 2014
Published: November 13, 2014

REFERENCES

Avdonin, V., and Hoshi, T. (2001). Modification of voltage-dependent gating of potassium channels by free form of tryptophan side chain. Biophys. J. *81*, 97–106.

Aziz, A., and Anderson, G.H. (2003). Exendin-4, a GLP-1 receptor agonist, interacts with proteins and their products of digestion to suppress food intake in rats. J. Nutr. *133*, 2326–2330.

Bansal, T., Alaniz, R.C., Wood, T.K., and Jayaraman, A. (2010). The bacterial signal indole increases epithelial-cell tight-junction resistance and attenuates indicators of inflammation. Proc. Natl. Acad. Sci. USA *107*, 228–233.

Berg, J., Hung, Y.P., and Yellen, G. (2009). A genetically encoded fluorescent reporter of ATP:ADP ratio. Nat. Methods *6*, 161–166.

Bryan, G.T. (1966). Urinary excretion of indoxyl sulfate (indican) by human subjects ingesting a semisynthetic diet containing variable quantities of L-tryptophan. Am. J. Clin. Nutr. *19*, 113–119.

Cani, P.D., Everard, A., and Duparc, T. (2013). Gut microbiota, enteroendocrine functions and metabolism. Curr. Opin. Pharmacol. *13*, 935–940.

Chimerel, C., Murray, A.J., Oldewurtel, E.R., Summers, D.K., and Keyser, U.F. (2013). The effect of bacterial signal indole on the electrical properties of lipid membranes. Chemphyschem *14*, 417–423.

DeMoss, R.D., and Moser, K. (1969). Tryptophanase in diverse bacterial species. J. Bacteriol. *98*, 167–171.

Devaraj, S., Hemarajata, P., and Versalovic, J. (2013). The human gut microbiome and body metabolism: implications for obesity and diabetes. Clin. Chem. *59*, 617–628.

Drucker, D.J., Jin, T., Asa, S.L., Young, T.A., and Brubaker, P.L. (1994). Activation of proglucagon gene transcription by protein kinase-A in a novel mouse enteroendocrine cell line. Mol. Endocrinol. *8*, 1646–1655.

Friedlander, R.S., Moss, C.E., Mace, J., Parker, H.E., Tolhurst, G., Habib, A.M., Wachten, S., Cooper, D.M., Gribble, F.M., and Reimann, F. (2011). Role of phosphodiesterase and adenylate cyclase isozymes in murine colonic glucagon-like peptide 1 secreting cells. Br. J. Pharmacol. *163*, 261–271.

Garber, A., Henry, R., Ratner, R., Garcia-Hernandez, P.A., Rodriguez-Pattzi, H., Olvera-Alvarez, I., Hale, P.M., Zdravkovic, M., and Bode, B.; LEAD-3 (Mono) Study Group (2009). Liraglutide versus glimepiride monotherapy for type 2 diabetes (LEAD-3 Mono): a randomised, 52-week, phase III, double-blind, parallel-treatment trial. Lancet *373*, 473–481.

Gong, F., Ito, K., Nakamura, Y., and Yanofsky, C. (2001). The mechanism of tryptophan induction of tryptophanase operon expression: tryptophan inhibits release factor-mediated cleavage of TnaC-peptidyl-tRNA(Pro). Proc. Natl. Acad. Sci. USA *98*, 8997–9001.

Gribble, F.M., Williams, L., Simpson, A.K., and Reimann, F. (2003). A novel glucose-sensing mechanism contributing to glucagon-like peptide-1 secretion from the GLUTag cell line. Diabetes *52*, 1147–1154.

Grynkiewicz, G., Poenie, M., and Tsien, R.Y. (1985). A new generation of Ca2+ indicators with greatly improved fluorescence properties. J. Biol. Chem. *260*, 3440–3450.

Karlin, D.A., Mastromarino, A.J., Jones, R.D., Stroehlein, J.R., and Lorentz, O. (1985). Fecal skatole and indole and breath methane and hydrogen in patients with large bowel polyps or cancer. J. Cancer Res. Clin. Oncol. *109*, 135–141.

Lee, J.H., and Lee, J. (2010). Indole as an intercellular signal in microbial communities. FEMS Microbiol. Rev. *34*, 426–444.

Marre, M., Shaw, J., Brändle, M., Bebakar, W.M., Kamaruddin, N.A., Strand, J., Zdravkovic, M., Le Thi, T.D., and Colagiuri, S.; LEAD-1 SU study group (2009). Liraglutide, a once-daily human GLP-1 analogue, added to a sulphonylurea over 26 weeks produces greater improvements in glycaemic and weight control compared with adding rosiglitazone or placebo in subjects with Type 2 diabetes (LEAD-1 SU). Diabet. Med. *26*, 268–278.

Mutch, D.M., Fuhrmann, J.C., Rein, D., Wiemer, J.C., Bouillot, J.L., Poitou, C., and Clément, K. (2009). Metabolite profiling identifies candidate markers reflecting the clinical adaptations associated with Roux-en-Y gastric bypass surgery. PLoS ONE *4*, e7905.

Nauck, M., Frid, A., Hermansen, K., Shah, N.S., Tankova, T., Mitha, I.H., Zdravkovic, M., Düring, M., and Matthews, D.R.; LEAD-2 Study Group (2009). Efficacy and safety comparison of liraglutide, glimepiride, and placebo, all in combination with metformin, in type 2 diabetes: the LEAD (liraglutide effect and action in diabetes)-2 study. Diabetes Care *32*, 84–90.

Nicholson, J.K., Holmes, E., Kinross, J., Burcelin, R., Gibson, G., Jia, W., and Pettersson, S. (2012). Host-gut microbiota metabolic interactions. Science *336*, 1262–1267.

Nyangale, E.P., Mottram, D.S., and Gibson, G.R. (2012). Gut microbial activity, implications for health and disease: the potential role of metabolite analysis. J. Proteome Res. *11*, 5573–5585.

Parker, H.E., Adriaenssens, A., Rogers, G., Richards, P., Koepsell, H., Reimann, F., and Gribble, F.M. (2012). Predominant role of active versus facilitative glucose transport for glucagon-like peptide-1 secretion. Diabetologia *55*, 2445–2455.

Peters, C.T., Choi, Y.H., Brubaker, P.L., and Anderson, G.H. (2001). A glucagon-like peptide-1 receptor agonist and an antagonist modify macronutrient selection by rats. J. Nutr. *131*, 2164–2170.

Petersen, N., Reimann, F., Bartfeld, S., Farin, H.F., Ringnalda, F.C., Vries, R.G., van den Brink, S., Clevers, H., Gribble, F.M., and de Koning, E.J. (2014). Generation of L cells in mouse and human small intestine organoids. Diabetes *63*, 410–420.

Piñero-Fernandez, S., Chimerel, C., Keyser, U.F., and Summers, D.K. (2011). Indole transport across *Escherichia coli* membranes. J. Bacteriol. *193*, 1793–1798.

Plaisancié, P., Dumoulin, V., Chayvialle, J.A., and Cuber, J.C. (1995). Luminal glucagon-like peptide-1(7-36) amide-releasing factors in the isolated vascularly perfused rat colon. J. Endocrinol. *145*, 521–526.

Psichas, A., Sleeth, M.L., Murphy, K.G., Brooks, L., Bewick, G., Hanyaloglu, A.C., Ghatei, M.A., Bloom, S.R., and Frost, G. (2014). The short chain fatty acid propionate stimulates GLP-1 and PYY secretion via free fatty acid receptor 2 in rodents. Int. J. Obes. (Lond.), Published online August 11, 2014. http://dx.doi.org/10.1038/ijo.2014.153.

Reimann, F., and Gribble, F.M. (2002). Glucose-sensing in glucagon-like peptide-1-secreting cells. Diabetes *51*, 2757–2763.

Reimann, F., Maziarz, M., Flock, G., Habib, A.M., Drucker, D.J., and Gribble, F.M. (2005). Characterization and functional role of voltage gated cation conductances in the glucagon-like peptide-1 secreting GLUTag cell line. J. Physiol. *563*, 161–175.

Reimann, F., Habib, A.M., Tolhurst, G., Parker, H.E., Rogers, G.J., and Gribble, F.M. (2008). Glucose sensing in L cells: a primary cell study. Cell Metab. *8*, 532–539.

Smith, E.A., and Macfarlane, G.T. (1996). Enumeration of human colonic bacteria producing phenolic and indolic compounds: effects of pH, carbohydrate availability and retention time on dissimilatory aromatic amino acid metabolism. J. Appl. Bacteriol. *81*, 288–302.

Tarasov, A.I., Semplici, F., Ravier, M.A., Bellomo, E.A., Pullen, T.J., Gilon, P., Sekler, I., Rizzuto, R., and Rutter, G.A. (2012). The mitochondrial Ca2+ uniporter MCU is essential for glucose-induced ATP increases in pancreatic β-cells. PLoS ONE *7*, e39722.

Tarini, J., and Wolever, T.M. (2010). The fermentable fibre inulin increases postprandial serum short-chain fatty acids and reduces free-fatty acids and ghrelin in healthy subjects. Appl. Physiol. Nutr. Metab. *35*, 9–16.

Tolhurst, G., Zheng, Y., Parker, H.E., Habib, A.M., Reimann, F., and Gribble, F.M. (2011). Glutamine triggers and potentiates glucagon-like peptide-1 secretion by raising cytosolic Ca2+ and cAMP. Endocrinology *152*, 405–413.

Tolhurst, G., Heffron, H., Lam, Y.S., Parker, H.E., Habib, A.M., Diakogiannaki, E., Cameron, J., Grosse, J., Reimann, F., and Gribble, F.M. (2012). Short-chain fatty acids stimulate glucagon-like peptide-1 secretion via the G-protein-coupled receptor FFAR2. Diabetes *61*, 364–371.

Wang, T.J., Larson, M.G., Vasan, R.S., Cheng, S., Rhee, E.P., McCabe, E., Lewis, G.D., Fox, C.S., Jacques, P.F., Fernandez, C., et al. (2011). Metabolite profiles and the risk of developing diabetes. Nat. Med. *17*, 448–453.

Zariwala, H.A., Borghuis, B.G., Hoogland, T.M., Madisen, L., Tian, L., De Zeeuw, C.I., Zeng, H., Looger, L.L., Svoboda, K., and Chen, T.W. (2012). A Cre-dependent GCaMP3 reporter mouse for neuronal imaging in vivo. J. Neurosci. *32*, 3131–3141.

Zinman, B., Gerich, J., Buse, J.B., Lewin, A., Schwartz, S., Raskin, P., Hale, P.M., Zdravkovic, M., and Blonde, L.; LEAD-4 Study Investigators (2009). Efficacy and safety of the human glucagon-like peptide-1 analog liraglutide in combination with metformin and thiazolidinedione in patients with type 2 diabetes (LEAD-4 Met+TZD). Diabetes Care *32*, 1224–1230.

Zuccato, E., Venturi, M., Di Leo, G., Colombo, L., Bertolo, C., Doldi, S.B., and Mussini, E. (1993). Role of bile acids and metabolic activity of colonic bacteria in increased risk of colon cancer after cholecystectomy. Dig. Dis. Sci. *38*, 514–519.

Cell Reports, Volume *9*

Supplemental Information

Bacterial Metabolite Indole Modulates Incretin Secretion from Intestinal Enteroendocrine L Cells

Catalin Chimerel, Edward Emery, David K. Summers, Ulrich Keyser, Fiona M. Gribble, and Frank Reimann

Supplementary Information

Bacterial metabolite indole modulates incretin secretion from intestinal enteroendocrine L-cells.

Catalin Chimerel, Edward Emery, David K. Summers, Ulrich Keyser, Fiona M. Gribble and Frank Reimann

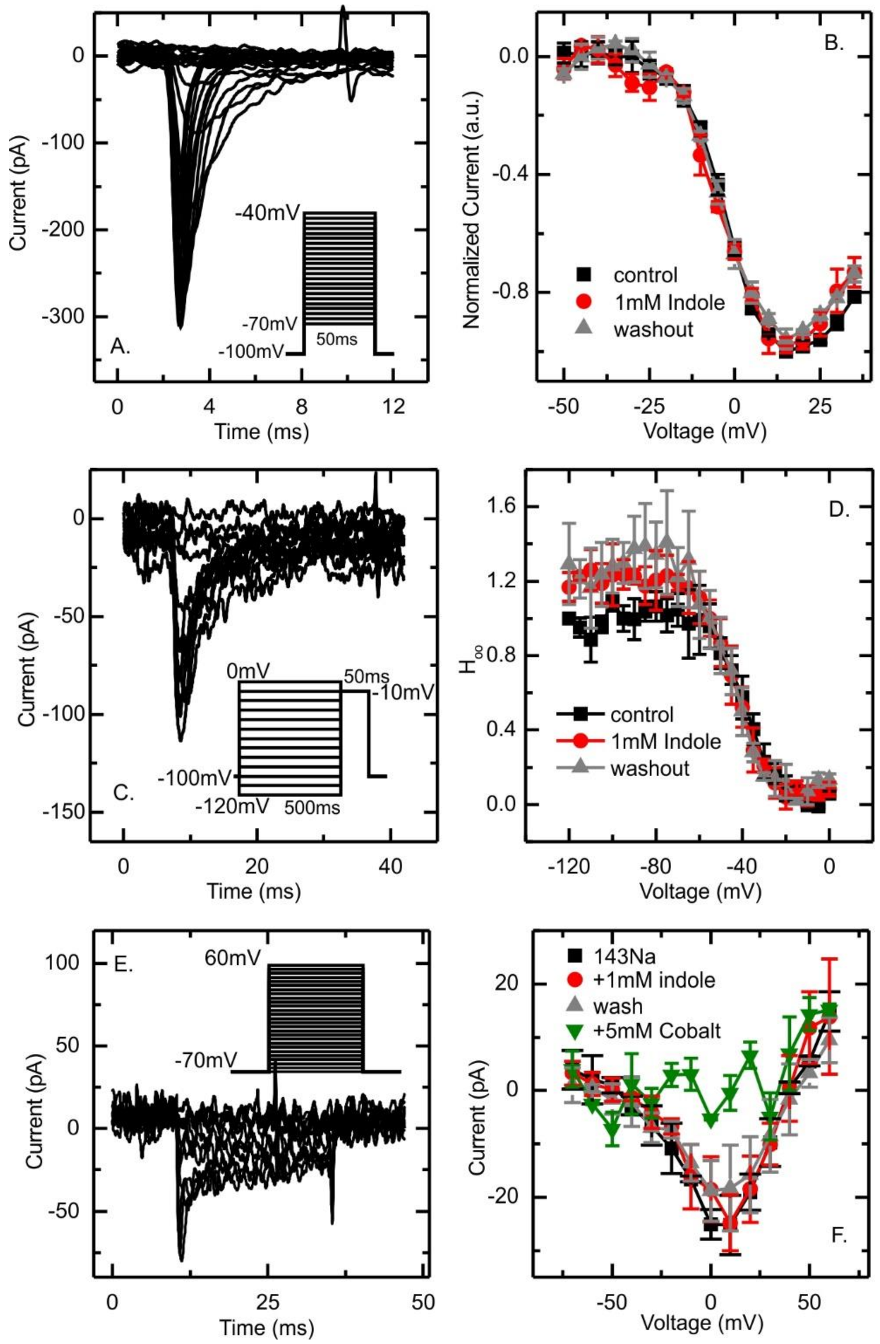

Supplementary Figure 1. **Effect of indole on sodium and calcium currents in GLUTag cells, related to Figure 2. A.** Current responses to 50 ms voltage steps of 5 mV increments were

applied from a holding potential of -100 mV (shown in the inset) applied in a whole cell voltage clamp recording in saline buffer containing inhibitors of voltage gated K^+ channels (see material and methods). Recordings were done in a whole cell voltage clamp recording where 50 ms voltage steps of 10 mV increments were applied from a holding potential of -100 mV. The raw data shown in panel A were down-sampled from 10 kHz to 1 kHz with a digital low pass filter (Clampfit 10.3.1.4). **B.** Current voltage relationship of the peak currents obtained when activating voltage gated Na^+ channels as in A. Each individual experiment was normalized to the maximum peak current at + 15 mV control. Data show are mean ± SEM of n=3 cells. **C.** Steady-state inactivation of Na^+ currents measured by holding the membrane at -100 mV for 500 ms at conditioning voltages between -120 and 0 mV (at 5 mV increments) before stepping to -10 mV for 50 ms and then to -100 mV. The inset shows the voltage pulse protocol. Only the current reponse to the test pulse to -10 mV is shown. The raw data shown in panel A were down-sampled from 10 kHz to 1 kHz with a digital low pass filter (Clampfit 10.3.1.4). An electrical interference Filter (Clampfit 10.3.1.4) was applied to remove the power line interference at 50 Hz. **D.** Mean peak currents from cells recorded as in C, normalized to the maximum peak current at -120 mV control for each individual experiment (H_∞), and plotted against the holding potential applied during the conditioning pules. Data show are mean ± SEM of N=3 cells. **E.** Current responses to 25 ms voltage steps of 5 mV increments, applied from a holding potential of -70 mV (shown in the inset), in a whole cell voltage clamp recording. Voltage gated Na^+ and K^+ current were blocked with specific inhibitors (see material and methods). The raw data shown in panel A were down-sampled from 10 kHz to 1 kHz with a digital low pass filter (Clampfit 10.3.1.4) . An electrical interference Filter (Clampfit 10.3.1.4) was applied to remove the power line interference at 50 Hz. **F.** Current voltage relationship of the peak currents obtained from cells recorded as in A (N=3).
